# Considering treatment-as-prevention scale-up for Australian prisons: a qualitative sub-study of expert stakeholders from the Australian ‘surveillance and treatment of prisoners with hepatitis C’ project (SToP-C)

**DOI:** 10.1186/s12954-021-00494-4

**Published:** 2021-04-26

**Authors:** Jake Rance, Lise Lafferty, Carla Treloar, the SToP-C Study Group, Stuart Loveday, Gregory Dore, Andrew Lloyd, Jason Grebely, Tony Butler, Natasha Martin, Georgina Chambers, Carla Treloar, Marianne Byrne, Roy Donnelly, Colette McGrath, Julia Bowman, Lee Trevethan, Luke Grant, Terry Murrell, Nicky Bath, Mary Harrod, Alison Churchill, Kate Pinnock, Sallie Cairnduff

**Affiliations:** Centre for Social Research in Health, University of New South Wales, Level 3, John Goodsell Building, Sydney, NSW 2052 Australia

**Keywords:** Australia, Prisons, Hepatitis C, Treatment-as-prevention, Implementation trial

## Abstract

**Background:**

With direct-acting antivirals dramatically reshaping the public health response to the hepatitis C virus (HCV), prisons are set to play a critical role in elimination efforts. Despite the theoretical demonstration of HCV treatment-as-prevention in prison in mathematical modeling, limited empirical data exist. The Australian ‘Surveillance and Treatment of Prisoners with Hepatitis C’ project (SToP-C) is the world’s first trial of HCV treatment-as-prevention in prison. Drawing on interviews with HCV expert stakeholders, this paper explores the factors respondents identified as crucial to the success of future scale-up. Accounting for such perspectives matters because of the influence expert discourse has in shaping implementation.

**Methods:**

Semi-structured interviews were conducted with nineteen HCV experts working across key policy, advocacy, research and clinical dimensions of the Australian HCV response. Data were coded using qualitative data management software (NVivo 11). Analysis proceeded via a hybrid deductive and inductive approach.

**Results:**

Notwithstanding concerns regarding the lack of primary prevention in Australian prisons, stakeholders reported broad levels of support for the intervention and for the future scale-up of HCV treatment. A number of considerations, both external and internal to the prison system, were identified as key. The principal external factor was an enabling political-cum-policy environment; internal factors included: obtaining support from prisons’ executive and custodial staff; promoting health within a security-first institutional culture; allocating time for treatment within prisoners’ tightly regulated schedules; ensuring institutional stability during treatment given the routine movement of prisoners between prisons; prioritizing the availability of retreatment given the paucity of primary prevention; and securing sufficient clinical space for treatment.

**Conclusion:**

The challenges to implementation are considerable, ranging from macrolevel concerns to in-prison logistical matters. Nonetheless, we argue that prisons remain an obvious setting for treatment scale-up, not only for prevention and potential elimination benefit, but for the treatment opportunities they afford a socially disadvantaged and underserved population. While noting widespread concerns among respondents regarding the paucity of primary prevention in Australian prisons, results indicate broad levels of support among expert stakeholders for HCV treatment scale-up in prison.

## Introduction

Due principally to the criminalization and imprisonment of people who use drugs, and the role of unsterile injecting equipment as the primary risk factor in incident cases, the global burden of hepatitis C (HCV) is disproportionately borne by people in prison [1–3]. Prevalence in custodial settings is up to 40 times greater than in the general community [2]. In Australia, while antibody prevalence among the general prison population is approximately 22%, this rises to well-over double that among prisoners who report a history of injecting drug use [4]. Although rates of injecting decrease following imprisonment [5], the frequency of sharing injecting equipment increases, thereby significantly raising the per episode risk of HCV transmission [6].

Within both community and custodial settings, the curative potential of direct-acting antivirals (DAAs) has inspired a dramatic reconceptualization in the public health approach: from one of prevention and chronic disease management to one of population-level treatment-as-prevention and viral elimination [7]. Given the high HCV prevalence and regular turnover among prisoners with histories of injecting drug use—eight out of ten report previous incarceration, including 66% in the past year [4]—custodial settings have been identified as critical sites for treatment scale-up [8]. In Australia, when the Commonwealth Government announced its commitment to fund universal access to DAAs in December 2015, specific provisions were included to ensure the prioritization of treatment for people in prison [9]. And in the following year, prisons were again included as a priority setting [10] with Australia signing up to the ambitious 2030 elimination targets set by the World Health Organisation [11].

Globally, micro-elimination [8] efforts in prison have been hampered by the limited and inconsistent provision of harm reduction measures—such as opioid treatment programs (OTP) and prison needle syringe programs (PNSP)—despite strong evidence for their effectiveness in reducing HCV transmission [12–14]. In Australian prisons, the primary blood-borne virus prevention measure is OTP; Fincol, a bleach alternative promoted for cleaning used injecting equipment, is only available in some jurisdictions [15]. Neither measure has been associated with a significant reduction in HCV incidence, with concerns raised regarding the adequate coverage of the former [14], and the efficacy and practicality of the latter [15]. Indeed, in early 2020 the Justice Health & Forensic Mental Health Network, the state-government agency responsible for prison-health service delivery in New South Wales (NSW), called for the increase and expansion of existing harm reduction measures, including the introduction of a PNSP. This call was made in response to rising rates of *in-prison* HCV reinfection following DAA treatment [14].

Against this backdrop, the advent of highly curative new treatments has catalyzed interest in a treatment-as-prevention approach in custodial settings. Prisoner populations’ high viral prevalence, combined with tight prison regulation and limited harm reduction measures make for an opportune and advantageous setting for a real-world trial [16, 17]. Despite demonstrations of its feasibility within mathematical modeling work [1, 18, 19], limited empirical data exist regarding HCV treatment-as-prevention in  real-world prison settings. The Australian ‘Surveillance and Treatment of Prisoners with Hepatitis C’ project (SToP-C) [20] is the world’s first implementation trial of HCV treatment scale-up *as* prevention in prison. Drawing on interview data collected from HCV expert stakeholders as part of the SToP-C qualitative sub-study, the aim of this article is to document those aspects of the trial, and of the prison system more broadly, identified by respondents as key to future treatment-as-prevention scale-up in prison.

While the finalized quantitative results of the trial are yet to be released, our qualitative sub-study offers valuable, standalone insights for those attempting to achieve population-health effects in treatment-as-prevention programs implemented outside trial conditions. Findings from our pre-treatment interviews with prisoners documented concerns regarding patient confidentiality and potential HCV-related stigma [21]. Despite broadly welcoming the opportunity for enhanced access to DAA-therapy, prisoners reported fears regarding reinfection in the context of limited places on OTP and the absence of PNSP [22]. These fears were again reiterated during post-treatment interviews [23], particularly among participants reporting ongoing injecting drug. Interviews with prison officers highlighted the critical role they played in facilitating study enrolment and engagement [24], emphasizing the importance of trust and non-judgment in relation to prisoner-patients. Finally, drawing on interview data collected from the cohort analyzed here, we explored expert stakeholder assessments regarding the acceptability of SToP-C’s treatment-as-prevention approach [25].

In this article, we examine interviews conducted with a range of HCV expert stakeholders external to the trial itself. Such expertise is critical, allowing for respondents to comment on and influence the implementation of scale-up *beyond* simply the SToP-C trial. It is important to note here that the ‘key considerations’ identified by respondents as crucial to successful scale-up reflect a combination of insights drawn both directly from the trial itself and from respondents knowledge of the Australian prison system more broadly. Following the implementation literature, we understand ‘scale-up’ as the deliberate effort ‘to increase the impact of successfully tested health interventions so as to benefit more people and to foster policy and program development on a lasting basis’ (26: 2).

## Methods^1^

SToP-C was conducted across four NSW public prisons between 2014–2019, including one women’s minimum-medium security facility, and one minimum plus two maximum security men’s facilities. The primary objective of the trial was to demonstrate the effectiveness of the rapid scale-up of testing and treatment in reducing hepatitis C incidence, and ultimately, the prevalence of active infection (i.e., treatment-as-prevention). The initial surveillance phase identified patients with current infection (HCV RNA positive) who were subsequently offered treatment. Ongoing surveillance evaluated the impact of treatment on new incidents, both primary and reinfection. Special provisions, including the employment of dedicated nurses and custodial officers as SToP-C ‘champion,’ along with the hosting of promotional BBQs for prisoners (plus giveaways such as sweets and hats), were funded as part of the trial.

A SToP-C qualitative sub-study of key stakeholders from both the study prisons (prisoners, prison officers and prison-health staff) and the community (expert stakeholders) was conducted. Interviews with prisoner participants were conducted both before and after treatment, while interviews with the other stakeholders were conducted once towards the end of the trial. This paper presents findings from interviews with HCV expert stakeholders.

Recruitment of expert stakeholders followed a purposive sampling method [26], with the study team, in consultation with the project steering committee, compiling a list of candidates on the basis of their professional expertise. ‘Expertise’ was determined in line with the official Australian HCV response, formalized in 1999 with the release of the first National Hepatitis C Strategy and built on a model of partnership and collaboration between government and the peak organizations representing affected communities, health professionals and researchers [27]. Candidates for our study were selected on the basis of their leadership and/or seniority within these key partnership organizations. Our sample constitutes a diverse range of HCV expertise; its size reflects the available pool of Australian HCV experts with knowledge of the prison system.

Twenty-one candidates were invited by email to participate; two candidates declined to respond. Financial incentives or compensation were not offered. During 2018, nineteen semi-structured interviews with expert stakeholders were conducted, either face-to-face or by telephone. All stakeholders provided written or verbal consent prior to interview. Interviews lasted between 30 to 90 min. Questions ranged from those regarding the general management of HCV in custodial settings to those focused on the SToP-C trial. The former covered topics such as the current state of HCV prevention measures in prison, knowledge of DAAs and the advantages afforded by in-prison treatment. The latter included questions concerning the potential role of treatment in prevention (including potential limitations), policy and practice implications of the SToP-C trial, and barriers and facilitators to scale-up. It is important to emphasize here that our selection criteria for expert stakeholders did not require an intimate familiarity with the SToP-C trial. Interviews were structured in such a manner that all respondents were able to draw upon their relevant expertise irrespective of their level of knowledge about the trial itself. While SToP-C was used as a concrete example of a treatment-as-prevention intervention in prison, respondents were  also invited to reflect more generally on the concept and its scalability within the context of Australian prisons.

Interviews were digitally recorded, professionally transcribed, checked for accuracy and de-identified. Analysis proceeded via a hybrid deductive and inductive approach [28]. A coding frame was constructed by the study team, comprising inductive themes drawn from the interview data, and deductive themes constructed via a synthesis of Milat’s [29] population-health ‘implementation guide’ and Sekhon’s [30] notion of ‘acceptability’. Applying this framework, the interview data were then organized with the aid of qualitative data management software, NVivo 11. During our analysis of the data, it became evident that there were a number of critical issues or ‘key considerations’ germane to scaling-up treatment within the prison system. In light of the SToP-C trial and the intention to rollout HCV TasP in prison, there were obvious merits to exploring this topic. Reading across those ‘nodes’ or coding themes most germane to the topic of treatment scale-up, the first author (JR) then identified and collated the recurrent themes upon which our analysis is built. Our results section is organized and presented under these key considerations.

Ensuring respondent anonymity and confidentiality posed critical challenges given the relatively small size of the Australian HCV field. Particular care has therefore been taken to de-identify and anonymize transcripts, and to remove all demographic information from respondent attributions [31]. Hence, respondents are described simply as ‘P1′, ‘P2′ and so forth. Ethics approval was obtained from the following Human Research Ethics Committees: Justice Health & Forensic Mental Health Network (G621/13); Corrective Services NSW (qualitative sub-study approval on April 5, 2016); Aboriginal Health and Medical Research Council of NSW (1253/17); and UNSW Sydney (HC15645).

## Results

Respondents included eight women and 11 men, ranging in age from 28 to 72 years and relevant professional experience from 5 to 32 years (with an average of just over 19 years). The majority of participants were based in NSW (*n* = 13), the location of the four STOP-C prisons; the remainder were based in other states and territories (*n* = 6). Seven respondents were employed in community-based organizations providing support and advocacy for people affected by hepatitis C, people who use illicit drugs, or people who have been (or are) incarcerated and their families. The remaining twelve were employed across a range of state health and correctional health departments, clinical settings and research institutes. Our sample included five CEOs, six Directors, one Deputy-Director, three Professors/Clinical Program Heads, two Senior Policy Analysts, one Senior Project Officer and one Program Coordinator.

### External considerations

#### Enabling strategic environment

Respondents identified a supportive strategic environment as one of the keys to a successful scale-up. In the Australian context, ensuring policy and political support is complicated by a federated polity comprising six states and two territories, each with its own legislative powers for the administration of criminal justice. Australia’s current national HCV strategy [27] designates prisoners as a priority population, with the cost of pharmaceuticals covered by the Federal Government’s universal access program, enabling in-prison prescribing through its specialist ‘S100′ scheme. Nonetheless, such support is not necessarily reflected within state and territorial jurisdictions. Crucially, it is state and territory government departments that remain financially responsible for any of the administrative, logistical and structural changes required for scale-up. Consequently, as respondents noted, the issue has to be ‘pushed at the state and territory level’ (P19), where the big challenge is the relevant departments ‘having sufficient resourcing and appetite to say that prison is a worthwhile recipient for investment if we’re to scale it up.’ (P7).

Respondents were acutely aware of the challenging politics surrounding prison budgets, especially when it concerns prisoner health rather than prison security: ‘Prisoners, they’re the lowest of the low. Governments hate spending money on prisoners unless they have to.’ (P3) Here, federation creates particular challenges, with heterogenous models of prison administration operating across the different jurisdictions:

I think places like Victoria and NSW are better suited to do it [treatment scale-up]. [In Queensland] each hospital and health service operate independently, and they control prison health, so there’s no central direction. (P16)

Reflecting this need for action at a state and territory level, documents such as South Australia’s ‘Prisoner Blood-Borne Virus Action Plan’ were cited as exemplars of enabling legislation: there is ‘ministerial commitment from Corrections and Health [in South Australia] to address hepatitis C and blood borne viruses generally’ (P5). Similarly, NSW was identified as a state with ‘a specific strategy in terms of elimination going prison to prison’ (P12). Here, HCV elimination targets have been introduced as part of prison governance, as a measurable (and thus ‘actionable’) key performance index. Respondents argued that what is possible within prison necessarily reflects broader levels of strategic prioritization and support:

[M]aintaining the [NSW] statewide focus on the broader goals of eliminating hep C will be important. If that falls away, our ability to support […] Justice Health in their practical attempts to address it are diminished as well. (P16)

### Internal considerations

#### Prison governance

We turn now from matters concerning the broader external environment to those internal to the prison system itself. While respondents highlighted prison-related differences across states and territories, they also emphasized heterogeneity *within* jurisdictions. As these two respondents explained: ‘what people don’t understand about working in prisons is how different each prison is and how each prison’s its own fiefdom almost: its own little kingdom.’ (P2); ‘There’ll be some individual issues within a prison, but there’s greater inequity I imagine from prison to prison.’ (P12). As respondents argued, given each prison is ‘like a world unto itself, managed quite separately’ (P17), then gaining the support of the governor and the management team becomes central to any scale-up effort:

The attitude of the management team within the prison [is crucial]: if you haven’t got a management team that’s engaged, they can sink your project before it starts. So, you’ve gotta have the understanding and the buy-in. (P7).

#### Custodial staff acceptance

While the imprimatur of governors and their management teams was identified as crucial, so too, respondents argued, was a level of basic acceptance for scale-up among correctional staff. Prison officers have the power to influence the effectiveness of a health intervention, either ensuring or obstructing prisoner-patients have access to clinical support. Regarding the SToP-C intervention, one respondent referred to a culture of ‘resentment’ (P14) that existed among officers, with others noting that this ‘culture’ had the potential to manifest as a reluctance among officers to support the use of ‘expensive therapies’ (P12) on (undeserving) inmates whose infections were deemed ‘self-inflicted’ (P12).

Despite these concerns, it is important to note that such fears were largely not borne out in practice. Considerable efforts were made to promote to correctional staff the benefits for *everyone* in reducing HCV prevalence within the prison environment. Respondents noted the decision of study prisons to recruit a prison officer as a dedicated SToP-C ‘champion’ to promote the intervention, not only to inmates but to their colleagues too. Perhaps as a reflection of such efforts, the stigma associated with injecting drug use ultimately appeared not to translate into opposition to HCV treatment:

[I]f prisoners are viewed as getting Fincol … to clean injecting equipment, then you’re gonna be a target for getting yourself searched or being watched. Whereas I don’t get the same impression about [HCV] treatment generally and most of the corrective services [officers] tend to be, once we explain [treatment-as-prevention], fairly positive. They can see the benefits both to the patients, the community and, of course, for themselves. (P1)

Indeed, the argument regarding the reduction of viral prevalence has proven ‘a definite winner’ (P5) with prison staff:

So, on the custodial side … they’re very worried not only about their own personal risk but the risk that they would carry home to the missus [sic] or the kids. So, [reducing prevalence] is a very compelling tool that we use to engage with the custodial staff. (P5)

Or, as this respondent puts it: ‘surely, from an occupational health and safety perspective, it just makes sense. You’ve got a cure that the Commonwealth [government] is paying for […] It’s not gonna cost Corrections [state-government department responsible for prisons] anything and it just makes things potentially a bit safer.’ (P9).

### Security as priority

The matter of acceptance among custodial staff sits within a broader institutional culture of *security-first*. In prison, security trumps all. There can be a ‘cancellation of appointments because of lockdowns or other security reasons’ (P8), with minimal notice and without recourse. Here, the rollout of a health intervention is not a ready fit: it threatens a potential fault line of cultures, values and priorities. Without providing explicit examples from the trial itself, the issue of prison workplace culture was nonetheless raised by a number of respondents: it formed the institutional bedrock upon which the health intervention was played out. In this context, the imperative to ensure the support and goodwill of governors, management teams and the custodial officers who effectively oversee the daily running of prison, is clear.

### Reinfection risk

Despite respondents’ unanimous, in-principle support for the universal rollout of new HCV therapies in Australian prisons, many were troubled by the lack of effective primary prevention. The absence of PNSP, for example—described by one respondent as the ‘gold standard’ (P1) of prevention—was consistently noted. The subtext of such concern, particularly in the context of treatment-as-prevention, was the risk of reinfection. As this respondent put it:

The prison is, in my opinion, the key priority to achieving elimination more broadly across the state and in the community […] [But] can we achieve elimination using a treatment-as-prevention approach alone or do we require access to other prevention tools? (P11)

Despite such concerns, responses to the issue of reinfection ranged considerably. For some respondents, while comprehensive primary prevention remained a desirable aspiration, reinfection was nevertheless a problem readily overcome with effective treatments and a pragmatic policy of retreatment:

[E]ven though it’s not optimum that we don’t have the harm reduction access that we do in the community, reinfection is easy to retreat and there is access to retreatment for reinfection. (P12)

As much as everyone would like needle and syringe programs in Australian prisons, that’s just not gonna happen anytime soon. But you can eradicate the virus from the prison just by treating everyone […] It’s like treating a strep sore throat. You’re given five to 10 days of penicillin and it’s gone. (P3)

For a minority of respondents, however, the issue of reinfection was a touchstone for their opposition to treatment-as-prevention more broadly. Arguing that such an approach prioritizes treatment over prevention, they advocated instead for *prevention*-as-prevention:

[T]o have [treatment] as the stand-alone role is wrong. […] [Prisoners] don’t have the means to prevent themselves from reinfection once they have cured their hep C through the DAA-treatment. So, you’ve only got half the picture. (P8)

[I]t feels a little bit like a second-best option […] Treatment-as-prevention seems to be an incredibly expensive and resource-heavy way of going about something that could probably be a bit simpler if they just actually implemented proper prevention strategies in prisons. (P2)

### Prisoner time and movement

Time and movement were also considered key to scale-up efforts: ‘Time is a premium in prison… I mean it sounds a bit ridiculous when you think, *Well, how hard can it be in prison* [to find time for treatment]*.* Actually, it can be incredibly hard.’ (P7). Prisoners’ time is tightly regimented, with strict limits placed on periods spent outside of cells. As the respondent continues: ‘The constraints of the core day … you’ve got programs that you need to attend, work that you need to attend, phone calls that you need to make to your nearest and dearest’ (P7). While new DAAs have significantly shortened the duration of treatment, respondents noted the imperative of not only finding sufficient time for treatment itself but also for building relationships with potential patients.

Prisoner movement was another feature of prison life that similarly challenged treatment scale-up:

If there’s one thing we’ve learnt from SToP-C it’s how dynamic the movement is, even in the context of maximum-security prisons […] People saw SToP-C as working with, in inverted commas, a *captive population*, and that would [make it] really easy. Go in there and treat all these people and stop transmission. But that is absolutely not the case. (P12)

While respondents acknowledged transience was also a challenge for treatment rollout in the community, the issue was amplified in prison. And as one respondent pointed out, this is a growing concern: ‘The actual movement of patients in and out of different prisons [happens] much more quickly now. Even in the last two years …’ (P1). Respondents argued for the need to ‘minimize any harm that might come from high turnover or high movement rates within the prisons’ (P16), insisting that ‘there needs to be an absolute commitment that people who commence on treatment, unless released of course, are able to be retained in the center in which treatment can be provided.’ (P7).

### Physical space

Alongside prisoner time and movement, physical space was another practical yet fundamental consideration for effective scale-up. As this respondent explained: ‘logistics like space: have you got enough space to accommodate a big hep C treatment program when they’ve got other competing demands around primary care and mental health, and broader drug and alcohol issues?’ (P12). Indeed, for one of the SToP-C prisons, the shortage of clinical space was an ongoing issue: ‘there’s been other challenges like clinic space […] despite all their [prison name] efforts, they’ve really not been able to get that one solved.’ (P1). With only so many rooms available within each clinic, and a number of competing health programs (dental, population and primary health, for example) vying for limited space, dedicating one room solely to HCV care may mean compromising another program. As this respondent put it: ‘Space is generally a premium, particularly in NSW jails which are old and small, and not purpose-built […] So, having access to space can be a real nightmare.’ (P7).

### Resourcing scale-up from trial to implementation conditions

Transposing the conditions, and by implication, the cost, of the SToP-C trial to broad-based scale-up was a point of contention among some respondents. Several questioned its reproducibility: ‘I don’t think it’s a great model […] very expensive’ (P1); ‘there was too much time and effort put in and too much money […] it’s not replicable.’(P3). Singled out as examples of trial innovations too costly to be part of a broader roll-out was the employment of dedicated SToP-C nurses and corrections officers, the hosting of BBQs for prisoners, and the provision of study visit payments: ‘those sort of things, ongoing, I think are a possible challenge without a significant amount of money’ (P1). For as this respondent pointed out, one of the critical challenges facing scale-up is ‘a clearly limited capacity through an existing number of resources and the number of nurses.’ (P8).

Nonetheless, while some SToP-C innovations may prove too costly for broader implementation, other innovations have already been adopted in NSW prisons beyond the trial. Initially, prisoners receiving DAAs were required to attend the prison clinic for daily supervised dosing; now, due to SToP-C, the majority are taking their medications in their cells: ‘a huge change [it] really opens up the overall capacity in terms of treatment.’ (P12). Ultimately, as another respondent pointed out: ‘It depends. It’s resource related. I think as long as it’s well-resourced, scalability is not an issue […] [I]f you scale-up with insufficient resources and then it falls over then that’s a really compromising factor.’ (P7).

## Discussion

Despite modeling demonstrating the theoretical possibility of HCV treatment-as-prevention [1, 18, 19], SToP-C is the world’s first study to examine whether this is achievable in a real-world setting. This article analyses interview data collected from Australian HCV expert stakeholders as part of SToP-C’s qualitative sub-study. Our aim has been to explore the factors respondents identified as crucial to the success of a systemwide scale-up. Notwithstanding the inherent complexities of moving from trial to real-world implementation, respondents identified a range of key considerations, both external and internal to the prison system. The establishment of an enabling politico-policy environment was identified as the principle external consideration. Key internal considerations included the importance of enlisting support from both custodial executive and floor staff; negotiating health in a security-first institutional culture; and securing not only *time* for treatment amid highly regulated prisoner timetables and the routine movement of inmates, but sufficient clinical *space*.

While there is a burgeoning health-related implementation literature [32–34], none-to-date addresses the scale-up of HCV treatment-as-prevention in prison. Although based on Australian data, our results are linked to policies, processes and practices beyond the immediacy of their setting and are thus of ‘theoretical generalizability’ [35] to prison-based HCV treatment scale-up efforts internationally. This is particularly so given the shared demographic and structural features of custodial settings worldwide: of the overrepresentation of people who inject drugs; the disproportionately high rates of HCV prevalence; the inconsistent provision or absence of primary prevention measures; the political disinclination to champion the health and welfare of prisoners; and the almost inevitable prioritization of penal security over prisoner welfare in matters of prison governance. With injecting drug use now acknowledged as a ‘normative characteristic’ [36] of incarceration worldwide, our findings have direct implications not only for people who inject drugs in prison but also for the broader community. Reducing HCV prevalence among prisoners inevitably reduces the risk of community transmission post-release.

While the challenges of translating the outcomes of health-related trials to real-world settings are well documented [33, 37], there is growing recognition of the ‘crucial’ [38] contribution qualitative research can make to healthcare interventions. Arguably, this is no more so than in the case of prisons where the challenges are unique. While interventions within general healthcare systems are required to demonstrate cost effectiveness, budgetary responsibility and so forth, they nonetheless tend to be working within a shared cultural logic and ethos of care. The prison system, however, is underpinned by an opposing logic and ethos: one of security, even punishment at times [39, 40]. In custodial settings, healthcare is marginalized, with the purview of medical professionals subordinated to the authority of prison administrations [38]. Given these complexities, an appreciation of the challenges arising during the SToP-C trial provides valuable lessons for future scale-up.

While the antagonism of logics outlined above was certainly evident early on during SToP-C, it was largely ameliorated through intensive educational efforts on the part of the study team. Explaining the epidemiology of the virus helped not only address some of the lingering myths surrounding transmission risks (such that HCV is airborne), but also assuage some of the resentment custodial staff initially felt towards the provision of an ‘expensive’ treatment for prisoners—by reducing viral prevalence among inmates it was made clear that workplace safety for all staff would be enhanced. On an operational level, the employment of a dedicated SToP-C ‘champion’ recruited from among existing custodial staff was another means of garnering investment in the trial among custodial staff.

Some of the other key challenges identified by our respondents were also resolved during the trial. It was decided that prisoner-patients being moved mid-treatment (to a non-study prison) would be handed over to the respective prison-health staff for follow-up as per standard of care, and their study medication transferred along with them. Prisoner-patients released mid-treatment were given their remaining supply of medication along with a letter for their general practitioner outlining the recommended follow-up tests. Similarly, a solution was found for ensuring adequate prisoner time and clinical space. Initially, inmates receiving treatment were escorted daily to the medical clinic for directly-observed therapy: a huge impost on both officers’ time and clinic space. Following an assessment of other health conditions, it was decided that the majority of prisoners could take responsibility for self-administration. Consequently, the majority of prisoner-patients took their DAAs daily in their own cells—an innovation subsequently adopted in other, non-study prisons. Here again, the provision of education for custodial (and health) staff was crucial. Once staff appreciate that DAAs were not ‘valuable’ targets for diversion (i.e., both widely available in the community and not psychotropic) they supported the measure.

Regarding the key external challenge: the establishment of an enabling strategic environment has to some extent already been enacted in Australia through the introduction of a universal ‘access for all’ program. This scheme not only specifies cost-free treatment for prisoners (with no limit on retreatment if required) but is supported by a national HCV strategy that identifies prisoners and prisons as priority populations and settings. Despite its promise, however, this promise of universal access is complicated in Australia by a federated system that requires state-level political and policy support, including the preparedness of state-health and correctional departments to underwrite the administrative and logistical costs of scale-up. Indeed, elsewhere, we found few jurisdictions had updated their HCV-related health or prisoner-health policies following the introduction of universal access [41]. Such differences in policy and political readiness suggest a potentially staggered implementation of treatment scale-up, with some jurisdictions taking a proactive approach while others lag behind.

While, as we argue above, there is generalizable value to our findings that transcend their immediate empirical setting, we also recognize that there are conditions particular to the Australian prison system, including SToP-C, which may have influenced these findings. Australia’s universal-access scheme affords generous treatment conditions for people in prison, enabling cost-free treatment and retreatment if required. In international contexts, where treatment eligibility may be restricted on the basis of disease progression and/or drug and alcohol use, or where treatment is not cost-free for patients, there are potentially different matters at stake regarding treatment scale-up. Australia’s federated system means that some of our respondents’ key considerations may not be neatly transferrable to jurisdictions with different governance structures. We also recognize that employing a purposive sampling method necessarily means retaining control over respondent selection, if not over the content of the interviews themselves. While we maintain that this choice of recruitment method was appropriate given the nature of our study, it may still have influenced our findings.

## Conclusion

While there are strong epidemiological and human rights arguments for the rollout of effective new HCV-therapies in prison, their successful scale-up is not guaranteed. This article has identified a number of key considerations or challenges to inform such efforts, both internal to the prison system, and external. Notwithstanding these challenges—and those inherent in moving from trial to real-world conditions—we maintain that prisons remain obvious places for DAA scale-up, not only for potential micro- and macro-elimination benefit, but for the treatment opportunities they afford a socially disadvantaged and underserved population. Results from our sub-study have identified critical elements to consider in future HCV treatment scale-up initiatives.

## Data Availability

The datasets used and/or analyzed during the current study are available from the corresponding author on reasonable request.

## Abbreviations

DAA: Direct-acting antivirals
HCV: Hepatitis C virus
SToP-C: Surveillance and treatment of prisoners with hepatitis C
OTP: Opioid treatment programs
PNSP: Prison needle syringe program

## Footnotes

1 The description of our approach also appears in another paper drawn from the same dataset (25).

## References

[1] Papaluca T, Thompson A (2018). HCV elimination: breaking down the barriers to prison based care. Hepatoma Res.

[2] Grebely J, Bruneau J, Bruggmann P, Harris M, Hickman M, Rhodes T (2017). Elimination of hepatitis C virus infection among PWID: the beginning of a new era of interferon-free DAA therapy. Int J Drug Policy.

[3] Dolan K, Wirtz AL, Moazen B, Ndeffo-mbah M, Galvani A, Kinner SA (2016). Global burden of HIV, viral hepatitis, and tuberculosis in prisoners and detainees. The Lancet.

[4] Butler T, Simpson M. National Prison Entrants’ Blood-borne Virus Survey Report 2004, 2007, 2010, 2013, and 2016. Kirby Institute (UNSW Sydney); 2017.

[5] Wright NM, Tompkins CN, Farragher TM (2015). Injecting drug use in prison: prevalence and implications for needle exchange policy. Int J Prison Health.

[6] Cunningham EB, Hajarizadeh B, Bretana NA, Amin J, Betz-Stablein B, Dore GJ (2017). Ongoing incident hepatitis C virus infection among people with a history of injecting drug use in an Australian prison setting, 2005–2014: the HITS-p study. J Viral Hepatitis.

[7] Wallace J, Richmond J, Ellard J, Power J, Lucke J. Eradicating hepatitis C: the need for a public health response. Global Public Health. 2017:1–11. 10.1080/17441692.2017.1342851 30012072

[8] Lazarus JV, Safreed-Harmon K, Thursz MR, Dillon JF, El-Sayed MH, Elsharkawy AM (2018). The micro-elimination approach to eliminating hepatitis C: strategic and operational considerations. Semin Liver Dis.

[9] Dore GJ, Hajarizadeh B (2018). Elimination of hepatitis C virus in Australia: laying the foundation. Infect Dis Clin N Am.

[10] Hajarizadeh B, Grebely J, Martinello M, Matthews GV, Lloyd AR, Dore GJ (2016). Hepatitis C treatment as prevention: evidence, feasibility, and challenges. Lancet Gastroenterol Hepatol.

[11] World Health Organisation (2016). Global health sector strategy on viral hepatitis 2016–2021: towards ending viral hepatitis.

[12] Lazarus JV, Safreed-Harmon K, Hetherington KL, Bromberg DJ, Ocampo D, Graf N (2018). Health outcomes for clients of needle and syringe programs in prisons. Epidemiol Rev.

[13] Platt L, Minozzi S, Reed J, Vickerman P, Hagan H, French C (2018). Needle and syringe programmes and opioid substitution therapy for preventing HCV transmission among people who inject drugs: findings from a cochrane review and meta-analysis. Addiction.

[14] Blogg J, Wright T, McGrath C, Clegg J. Hepatitis C reinfection in prisons with broad access to direct acting antiviral treatments. Int J Drug Policy. 2020:102597. 10.1016/j.drugpo.2019.11.005 31952886

[15] Luciani F, Bretaña N, Teutsch S, Amin J, Topp L, Dore G. A prospective study of hepatitis C incidence in Australian prisoners. Addiction. 2014;109. 10.1111/add.12643 24916002

[16] Lazarus JV, Wiktor S, Colombo M, Thursz M (2017). Micro-elimination—a path to global elimination of hepatitis C. J Hepatol.

[17] Bartlett SR, Fox P, Cabatingan H, Jaros A, Gorton C, Lewis R (2018). Demonstration of near-elimination of hepatitis C virus among a prison population: The Lotus Glen Correctional Centre Hepatitis C Treatment Project. Clin Infect Dis.

[18] Bretaña NA, Gray RR, Cunningham EB, Betz-Stablein B, Ribeiro R, Graw F, et al. Combined treatment and prevention strategies for hepatitis C virus elimination in the prisons in New South Wales: a modelling study. Addiction. 10.1111/add.14830 31633853

[19] Dalgic OO, Samur S, Spaulding AC, Llerena S, Cobo C, Ayer T (2019). Improved health outcomes from hepatitis C treatment scale-up in Spain’s prisons: a cost-effectiveness study. Sci Rep.

[20] The Kirby Institute. Surveillance and Treatment of Prisoners With Hepatitis C (SToP-C) ClinicalTrials.gov: U.S. National Library of Medicine; 2019 [Available from: https://clinicaltrials.gov/ct2/show/NCT02064049?term=Prison&cond=Hepatitis+C&cntry=AU&draw=2&rank=2.

[21] Rance J, Lafferty L, Treloar C. ‘Behind closed doors, no one sees, no one knows’: hepatitis C, stigma and treatment-as-prevention in prison. Crit Public Health. 2018:1–11.

[22] Lafferty L, Rance J, Grebely J, Lloyd AR, Dore GJ, Treloar C (2018). Understanding facilitators and barriers of direct-acting antiviral therapy for hepatitis C virus infection in prison. J Viral Hepat.

[23] Lafferty L, Rance J, Grebely J, Dore GJ, Lloyd AR, Treloar C (2020). Perceptions and concerns of hepatitis C reinfection following prison-wide treatment scale-up: counterpublic health amid hepatitis C treatment as prevention efforts in the prison setting. Int J Drug Policy.

[24] Lafferty L, Rance J, Dore GJ, Lloyd AR, Treloar C. The role of social capital in facilitating hepatitis C treatment scale-up within a treatment-as-prevention trial in the male prison setting. Addiction. 10.1111/add.15277 33006784

[25] Rance J, Lafferty L, Treloar C. Expert stakeholder perspectives on the acceptability of treatment-as-prevention in prison: A qualitative sub-study of the ‘Surveillance and Treatment of Prisoners with Hepatitis C’ project (SToP-C) Addiction. (Accepted). 10.1111/add.15477 33751739

[26] Robinson OC (2014). Sampling in interview-based qualitative research: a theoretical and practical guide. Qual Res Psychol.

[27] Australian Government Department of Health and Ageing (2019). Fifth national hepatitis C strategy 2018–2022.

[28] Fereday J, Muir-Cochrane E (2006). Demonstrating rigor using thematic analysis: a hybrid approach of inductive and deductive coding and theme development. Int J Qual Methods.

[29] Milat A, Newson R, King L (2014). Increasing the scale of population health interventions: a guide.

[30] Sekhon M, Cartwright M, Francis JJ (2017). Acceptability of healthcare interventions: an overview of reviews and development of a theoretical framework. BMC Health Serv Res.

[31] Lancaster K (2017). Confidentiality, anonymity and power relations in elite interviewing: conducting qualitative policy research in a politicised domain. Int J Soc Res Methodol.

[32] May C (2013). Agency and implementation: Understanding the embedding of healthcare innovations in practice. Soc Sci Med.

[33] Glasgow RE, Lichtenstein E, Marcus AC (2003). Why don't we see more translation of health promotion research to practice? Rethinking the efficacy-to-effectiveness transition. Am J Public Health.

[34] Peters DH, Adam T, Alonge O, Agyepong IA, Tran N (2013). Implementation research: what it is and how to do it. BMJ.

[35] Neale J, Hunt G, Lankenau S, Mayock P, Miller P, Sheridan J (2013). Addiction journal is committed to publishing qualitative research. Addiction.

[36] Bonnycastle KD. Injecting risk into prison sentences: a quantitative analysis of a prisoner-driven survey to measure HCV/HIV seroprevalence, risk practices and viral testing at one Canadian male federal prison. Prison J. 2011;91.

[37] Zomahoun HTV, Ben Charif A, Freitas A, Garvelink MM, Menear M, Dugas M (2019). The pitfalls of scaling up evidence-based interventions in health. Glob Health Action.

[38] Maher L, Neale J (2019). Adding quality to quantity in randomized controlled trials of addiction prevention and treatment: a new framework to facilitate the integration of qualitative research. Addiction.

[39] Pont J, Stöver H, Wolff H (2012). Dual loyalty in prison health care. Am J Public Health.

[40] Allen SA, Aburabi R (2016). When security and medicine missions conflict: confidentiality in prison settings. Int J Prison Health.

[41] Lafferty L, Wild TC, Rance J, Treloar C (2018). A policy analysis exploring hepatitis C risk, prevention, testing, treatment and reinfection within Australia’s prisons. Harm Reduct J.

